# Photon-Counting Detector Computed Tomography and Hepatocellular Carcinoma—A Systematic Review of the Current Evidence

**DOI:** 10.3390/diagnostics16050743

**Published:** 2026-03-02

**Authors:** Salvatore Claudio Fanni, Francesco Damone, Markos Korakas, Riccardo Lencioni, Maurizia Rossana Brunetto, Emanuele Neri, Dania Cioni, Salvatore Masala, Mariano Scaglione

**Affiliations:** 1 Department of Translational Research, Academic Radiology, University of Pisa, 56126 Pisa, Italy; m.korakas@studenti.unipi.it (M.K.); riccardo.lencioni@unipi.it (R.L.); emanuele.neri@unipi.it (E.N.); dania.cioni@unipi.it (D.C.); 2 Department of Clinical and Experimental Medicine, University of Pisa, and the Hepatology Unit, Pisa University Hospital, 56124 Pisa, Italy; f.damone@studenti.unipi.it (F.D.); maurizia.brunetto@unipi.it (M.R.B.); 3 Radiology Department of Surgery, Medicine and Pharmacy, University of Sassari, Viale S. Pietro, 07100 Sassari, Italy; samasala@uniss.it (S.M.); mscaglione@uniss.it (M.S.); 4 Department of Radiology, James Cook University Hospital & Teesside University, Marton Road Marton Rd., Middlesbrough TS4 3BW, UK

**Keywords:** photon-counting detector computed tomography, hepatocellular carcinoma, diagnosis, virtual monoenergetic imaging, quantum iterative reconstruction

## Abstract

**Objectives:** The aim of this systematic review was to evaluate the current evidence on photon-counting detector computed tomography (PCCT) in hepatocellular carcinoma (HCC) imaging. **Methods:** A systematic literature search was performed in PubMed and Scopus, and five articles were finally included. **Results:** Four studies focused on the optimization of acquisition and reconstruction parameters such as slice thickness, kernels, virtual monoenergetic imaging (VMI), and quantum iterative reconstruction (QIR), with 50 keV reconstructions consistently associated with improved lesion conspicuity. QIR demonstrated significant noise reduction compared with filtered back projection, enhancing overall image quality, while one proof-of-concept study investigated dual-contrast PCCT, showing feasibility for simultaneous arterial and portal-phase acquisition. According to QUADAS-2, most studies presented a low or unclear risk of bias, with only one study rated at high risk for patient selection. **Conclusions:** In conclusion, PCCT shows promising technical advances and potential for improved HCC detection and characterization. Current evidence remains preliminary and focused on image quality rather than clinical outcomes; PCCT applications in routine practice are still largely unexplored.

## 1. Introduction

Hepatocellular carcinoma (HCC) is the most frequent primary liver malignancy, accounting for approximately 90% of primary liver cancer cases, and it represents a major global health burden. HCC remains among the leading causes of cancer-related mortality worldwide, with nearly 900,000 new diagnoses and more than 800,000 deaths estimated each year [1,2]. Despite improvements in prevention, surveillance strategies, and therapeutic options, outcomes remain poor at the population level, with a global 5-year survival rate generally below 20%. This unfavorable prognosis largely reflects the fact that a substantial proportion of patients are still diagnosed at an advanced stage, when curative-intent therapies are no longer feasible. By contrast, when HCC is detected early and treated with potentially curative approaches, survival can rise to 60–70%, emphasizing that timely detection is one of the strongest determinants of patient outcome [3]. Most HCCs develop in the setting of chronic liver disease. Historically, hepatitis B and C infections and alcohol-related liver disease have represented the predominant etiologic factors; however, the epidemiology of HCC is evolving, with an increasing contribution from metabolic dysfunction, i.e., associated steatotic liver disease and related metabolic comorbidities. Liver cirrhosis is present in approximately 80–90% of patients at diagnosis [4,5], and it fundamentally shapes both the biology of hepatocarcinogenesis and the diagnostic pathway, because the cirrhotic liver is characterized by heterogeneous parenchymal remodeling and the frequent presence of benign nodules that can complicate image interpretation. In clinical practice, delays in diagnosis remain common and translate into missed opportunities for surgical resection, liver transplantation, or local ablative therapies, which are predominantly applicable in early-stage disease.

HCC is unique among solid tumors as, in appropriate clinical contexts, a definitive diagnosis can often be established non-invasively based on imaging alone, without histological confirmation [6]. This paradigm relies on the characteristic vascular behavior of HCC, typically assessed with multiphasic contrast-enhanced CT, contrast-enhanced ultrasound (CEUS), or MRI. Surveillance in at-risk populations is commonly performed with abdominal ultrasound at six-month intervals, yet adherence to surveillance programs is frequently suboptimal, and test performance may be reduced in patients with obesity, advanced cirrhosis, or technically limited examinations. As a consequence, many patients continue to present with advanced disease, which is associated with a median survival of less than one year in several real-world settings [6]. Therefore, improving early detection with imaging strategies that are both sensitive and operationally reliable remains a central goal in HCC management [7]. Imaging is pivotal not only for detection and diagnosis but also for lesion characterization, staging, treatment allocation, and post-therapeutic follow-up [8]. Standardized diagnostic systems such as the Liver Imaging Reporting and Data System (LI-RADS) provide structured categorization of liver observations based on imaging features, enabling consistent communication and often reducing the need for invasive diagnostic procedures in patients with underlying cirrhosis [8,9,10]. Nonetheless, a major unmet clinical need persists for reliable detection and confident characterization of small or borderline lesions in cirrhotic livers, as well as a reduction in indeterminate categories (e.g., observations that remain in a “gray zone” with unclear management implications). In this setting, incremental improvements in image quality and quantitative robustness may translate into clinically meaningful changes, including fewer equivocal diagnoses and faster treatment decisions. Conventional imaging modalities continue to face important limitations. The sensitivity of CT decreases for sub-centimeter lesions, and benign cirrhotic nodules, i.e., regenerative or dysplastic, may partially mimic the enhancement patterns of HCC, leading to equivocal findings or indeterminate categorization [8]. CT is also affected by beam-hardening artifacts, electronic noise, and limited soft-tissue contrast, which can impair the assessment of tumor vascularity and restrict robust quantification of iodine uptake and fibrosis-related changes [11]. MRI generally provides higher sensitivity for small or early-stage lesions, but its use may be constrained by higher costs, longer acquisition times, limited availability, and contraindications in selected patients (including those with severe renal dysfunction or certain implanted devices). CEUS can be valuable in selected scenarios but is operator-dependent and may show reduced sensitivity in obese patients or in those with advanced cirrhosis [12]. In recent years, CT technology has evolved to address some of these limitations. Dual-energy CT (DECT) enables virtual monoenergetic reconstructions, iodine maps, and quantification of fat or iron content, potentially improving lesion conspicuity and characterization while allowing dose optimization [13]. Still, DECT may be affected by detector cross-talk, reduced spatial resolution at lower energies, and vendor-dependent variability in acquisition and reconstruction methods, which can hinder standardization and quantitative comparability across platforms and centers [14]. Photon-counting detector CT (PCCT) represents a further step in CT evolution. Unlike conventional energy-integrating detectors, photon-counting detectors directly measure the energy of individual X-ray photons, enabling true spectral imaging at the pixel level while substantially reducing electronic noise [15]. These properties translate into improved spatial and contrast resolution, fewer artifacts, enhanced multi-energy capabilities, and more reliable tissue and iodine quantification, often achievable at lower radiation doses [16,17]. From a clinical standpoint, these features are particularly relevant in HCC, where diagnostic confidence frequently hinges on subtle differences in enhancement and on the conspicuity of small lesions against a heterogeneous cirrhotic background. Following regulatory approval in 2021, PCCT systems are progressively being introduced into selected clinical environments [18]. Nevertheless, evidence supporting PCCT specifically in HCC imaging remains limited and fragmented, with available studies often characterized by heterogeneous designs, small cohorts, non-uniform reference standards, and a predominance of technical rather than patient-centered endpoints.

The aim of this systematic review is therefore to synthesize and critically appraise the available evidence on the role of photon-counting detector CT in the diagnosis and characterization of hepatocellular carcinoma, with a focus on current technical capabilities, methodological limitations, and future research directions.

## 2. Materials and Methods

### 2.1. Literature Search

This systematic review was conducted in accordance with the Preferred Reporting Items for the Systematic Reviews and Meta-Analyses (PRISMA) 2020 statement [19]. The completed PRISMA checklist is provided in the Supplementary Materials. Two reviewers (S.C.F. and F.D.) independently conducted a systematic literature search to identify all potentially relevant articles on two electronic databases, i.e., PubMed and Scopus. The following search string was adopted to identify all relevant articles: (“photon counting CT” OR “photon-counting CT” OR “photon counting computed tomography” OR “photon-counting computed tomography” OR “photon-counting detector CT” OR “photon-counting detector computed tomography” OR “PCCT”) AND(“hepatocellular carcinoma” OR “HCC” OR “liver cancer” OR “liver neoplasm*” OR “hepatic tumor*” OR “liver tumor*”). The last search was performed on 01 August 2025. No language, publication date, or study design filters were applied during the systematic search to maximize sensitivity and minimize the risk of missing relevant studies, given the emerging nature of PCCT technology. Systematic search results were exported in CSV and uploaded to Rayyan, a cloud-based platform specifically designed to support systematic review screening and collaboration [20].

### 2.2. Study Selection and Eligibility Criteria

After automated duplicate removal in Rayyan, two reviewers (F.D. and M.K.) independently and manually screened all retrieved records by title and abstract. Studies considered potentially eligible were subsequently assessed by full text. All the disagreements between the two reviewers were addressed by a third experienced reviewer (S.C.F.). Studies were included if they met any of the following criteria: (i) original research articles that investigated PCCT applications in the HCC clinical setting; (ii) studies involving human subjects with known or suspected HCC undergoing PCCT examination; (iii) studies reporting qualitative or quantitative imaging outcomes related to HCC detection, characterization, or image quality. Exclusion criteria included (i) animal studies and phantom-only studies without direct clinical correlation; (ii) case reports, systematic reviews, scoping or narrative reviews, editorials, commentaries, letters to the editor, conference abstracts, proceedings, books, and book chapters; (iii) studies not specifically addressing HCC or not applying PCCT technology. These criteria were chosen to ensure that only original investigations providing primary data relevant to PCCT applications in HCC were included.

### 2.3. Data Extraction

Data extraction was performed by two reviewers (S.C.F. and M.K.) using a predefined standardized data collection form. Extracted variables included first author, year of publication, study design, study aim, patient population, imaging protocol details, reconstruction techniques, reference standards, and main qualitative or quantitative outcomes. Given the heterogeneity of study designs, imaging protocols, outcome measures, and the predominantly technical nature of the included studies, a quantitative meta-analysis was not considered appropriate. Therefore, results were synthesized using a qualitative narrative approach, with findings grouped according to major thematic domains, including optimization of acquisition parameters, reconstruction strategies, and spectral imaging applications.

### 2.4. Qualitative Assessment

The Quality Assessment of Diagnostic Accuracy Studies-2 (QUADAS-2) tool was used to systematically evaluate the risk of bias and the applicability of the included studies [21]. This tool, widely regarded for evaluating the methodological quality of diagnostic accuracy research, assesses four key domains: (D1) patient selection, (D2) index test, (D3) reference standard, and (D4) flow and timing. Within each domain, risk of bias and concerns regarding applicability to the research question were independently judged by two reviewers (F.D. and M.K.), with disagreements resolved by consensus with a third reviewer (S.C.F.). Each item was rated as low risk, high risk, or some concerns, with the latter category applied when reporting was insufficient for a definitive judgment. This structured process ensured a transparent and reproducible assessment of study quality.

## 3. Results

**Figure 1:**
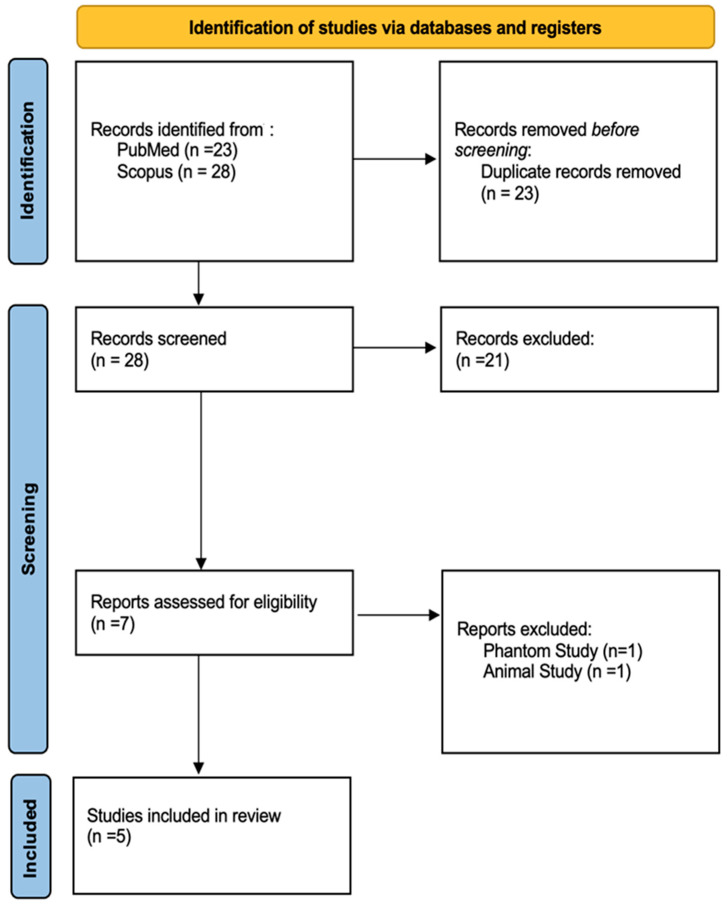
The study selection process flow diagram according to the PRISMA statement 2020 [19].

The systematic literature search identified a total of 51 records across the two investigated electronic databases. After the automated exclusion of duplicates (*n* = 23), 28 unique records were screened based on title and abstract. According to the inclusion and exclusion criteria, 21 records were excluded after title or abstract screening. Seven full-text articles were assessed; two were excluded (one was a phantom-only study and one was an animal study), resulting in a total of 5 articles included in the systematic review for further analysis. The study selection process is summarized in the PRISMA flowchart presented in Figure 1.

The characteristics of the included articles are summarized in Table 1.

The majority of the papers were published after 2023, with only one published before in 2017. Four studies were clinical studies conducted in patient populations with HCC, investigating optimization of PCCT acquisition protocols for HCC detection. The authors systematically varied acquisition and reconstruction parameters, including reconstruction kernels, VMI energy levels, slice thickness, imaging phases, and reconstruction algorithms (e.g., filtered back projection vs. quantum iterative reconstruction). One proof-of-concept study was included. In this study, the authors investigated the potential role of spectral PCCT for simultaneous dual-contrast multi-phase liver imaging by subsequently administering two contrast media, i.e., a gadolinium-based contrast agent and an iodine-based contrast agent. Among the clinical studies, sample sizes ranged from 24 to 49 patients, with a mean ± standard deviation of 35.5 ± 12.9 patients with viable HCC. One study specifically included patients with liver cirrhosis and HCC lesions, while the remaining clinical studies enrolled patients with known or suspected HCC undergoing diagnostic imaging. Reference standards varied across studies. Two of the five studies used a clinical reference standard based on MRI or CT follow-up; in one of these, histopathology was also available for confirmation of metastases/HCC. The remaining studies focused primarily on internal comparison of image quality metrics across different acquisition or reconstruction settings and did not employ an external diagnostic reference standard.

All clinical studies employed contrast-enhanced PCCT protocols, with a primary focus on arterial-phase imaging. One study compared arterial-phase and portal venous-phase acquisitions, while another implemented a triple-phase protocol. Acquisition parameters varied across studies, reflecting differences in scanner settings and study aims. Virtual monoenergetic images (VMI) were reconstructed across a range of energy levels, most commonly between 40 and 70 keV. Several studies reported results specifically at 50 keV, which was the most frequently evaluated energy level. Polychromatic image reconstructions were also included in selected studies for comparison purposes. Four studies investigated the impact of reconstruction parameters on image quality in PCCT imaging of HCC. Reconstruction kernels with varying degrees of sharpness were evaluated, including both softer and sharper kernels. Slice thicknesses ranged from ultra-thin reconstructions (0.4 mm) to thicker sections up to 3 mm. Image quality assessment methods included both quantitative metrics, such as image noise and contrast-to-noise ratio, and qualitative evaluations performed by expert readers. One study specifically assessed the effect of different reconstruction algorithms, comparing filtered back projection with multiple levels of quantum iterative reconstruction. Image quality outcomes were evaluated across reconstruction levels, with comparisons performed within each dataset.

The proof-of-concept study explored the feasibility of spectral PCCT for dual-contrast liver imaging. This investigation employed sequential administration of gadolinium-based and iodine-based contrast agents, corresponding to portal venous and arterial phases, respectively. Using numerical spectral PCCT data, the study assessed the ability to differentiate contrast materials based on their distinct energy-dependent attenuation properties, enabling separation of arterial and portal venous information within a single acquisition.

**Figure 2:**
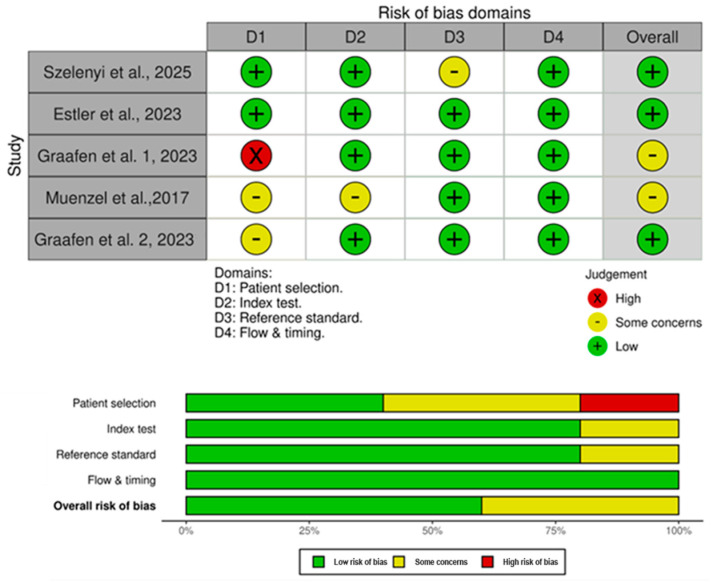
Risk of bias domains and histograms according to QUADAS-2 [27] for included papers [22,23,24,25,26].

The risk of bias, according to the QUADAS-2 evaluation, is summarized in Figure 2. All studies were rated as low risk regarding the D4, i.e., flow and timing. Four studies out of five were rated as low risk for both D2 and D3, i.e., index test and reference start, with the remaining being rated as unclear. In D1, regarding patient selection, one study was rated as high risk of bias due to missing inclusion and exclusion criteria.

## 4. Discussion

### 4.1. Current Evidence and Technical Focus of PCCT in HCC Imaging

PCCT is an emerging technology with multiparametric capabilities that has the potential to reshape hepatocellular carcinoma imaging [17]. Compared with conventional CT, it offers improved spatial and spectral resolution together with the possibility of radiation dose reduction [28]. Despite these theoretical advantages, its role in HCC imaging remains largely unexplored, and the available literature is still at a very early stage. The five studies included in this systematic review predominantly addressed technical aspects of acquisition and reconstruction, indicating that research in this field is currently focused on parameter optimization rather than on validated clinical applications. This pattern is consistent with the developmental trajectory of many emerging imaging technologies, in which technical refinement precedes widespread diagnostic validation. Accordingly, the current evidence on PCCT in HCC imaging is limited in both volume and scope and remains largely exploratory. All available studies were conducted in controlled research settings and were primarily designed to evaluate technical performance and image quality–related metrics, including image noise, contrast-to-noise ratio, lesion conspicuity, and the impact of different reconstruction strategies. In contrast, clinically oriented endpoints, such as diagnostic accuracy, inter-reader agreement, and effects on patient management, have not yet been systematically investigated. Furthermore, substantial heterogeneity exists across studies with respect to acquisition protocols, reconstruction parameters, energy level selection, and reference standards, reflecting the absence of standardized PCCT workflows for liver imaging and underscoring the early phase of technological adoption. As a result, direct comparison across studies remains challenging, and the available evidence should be interpreted primarily as a demonstration of technical feasibility rather than as validation of clinical effectiveness. In the specific context of HCC imaging, where subtle enhancement differences, cirrhotic background alterations, and small lesion size represent major diagnostic challenges, the translation of technical image quality improvements into meaningful clinical benefit has yet to be demonstrated. Consequently, photon-counting detector CT should currently be regarded as a promising research tool rather than a mature clinical modality, pending robust evidence from prospective studies employing standardized protocols and clinically relevant endpoints.

### 4.2. Optimization of Acquisition and Reconstruction Parameters

In their first study, Graafen et al. investigated the effect of sharpness levels on image quality at 50 keV and reconstruction algorithms in HCC patients. The authors reported that the lowest sharpness level (36) was associated with reduced noise (18 ± 2, *p* < 0.001) and improved lesion conspicuity. By contrast, higher sharpness levels increased noise, potentially compromising diagnostic quality. These findings highlight the importance of balancing spatial resolution and image noise, a longstanding challenge in liver CT imaging, particularly for the detection of small hypervascular lesions [29,30,31]. In a subsequent study, the same group investigated reconstruction algorithms, focusing on quantum iterative reconstruction (QIR). A progressive improvement in image quality was observed from QIR-1 to QIR-4, with QIR-4 achieving the greatest noise suppression (−67% vs. filtered back projection), together with superior lesion conspicuity [26]. These results were consistent with phantom experiments by Racine et al., who reported that PCCT combined with QIR enhanced focal liver lesion detection, particularly at low radiation doses, compared to conventional CT [29].

Szelenyi et al. further explored the interaction between slice thickness and reconstruction kernel selection in arterial-phase PCCT imaging. In their study, Br40 reconstructed at a 3 mm slice thickness served as the clinical reference. Compared with this setting, Br40 reconstructed at 1 mm provided significantly superior overall image quality, reflecting an improved balance between spatial resolution and image noise. Sharper kernels reconstructed at 1 mm (Br44 and Br48) were rated superior for hepatic vasculature assessment; however, the associated increase in image noise limited their utility for overall image quality assessment and did not result in a significant advantage over the reference reconstruction [25]. These observations are in line with broader evidence indicating that thinner slice reconstructions improve spatial resolution but require robust noise suppression to preserve diagnostic quality [31]. Notably, both Graafen et al. and Szelenyi et al. reconstructed images at 50 keV, a level widely regarded as optimal for abdominopelvic imaging [30,31].

Collectively, these studies indicate that PCCT performance in HCC imaging is highly dependent on protocol optimization and that improvements in lesion conspicuity are achieved not through maximal spatial resolution alone, but through a careful balance between reconstruction sharpness, slice thickness, energy level selection, and noise control strategies. Importantly, the available evidence is primarily derived from studies designed to assess technical image quality rather than diagnostic accuracy, and the clinical impact of these optimizations therefore remains to be established.

### 4.3. Spectral Imaging, Multiphasic Protocols and Potential Dose Implications

Beyond acquisition refinements, Estler et al. explored the diagnostic impact of different virtual monoenergetic images (VMIs, 40–70 keV) and compared arterial with portal-phase imaging. Although they found excellent diagnostic performance at 40 keV, arterial-phase acquisition increased radiation dose without clear diagnostic benefit, raising once more the clinical dilemma of multiphasic CT in cirrhotic patients [23]. Capturing the enhancement dynamics of HCC is essential for non-invasive diagnosis, but multiphasic protocols increase radiation, exam duration, and are susceptible to motion artifacts, especially in cirrhotic patients with compromised breath-hold capacity [8]. In this context, the spectral capabilities of advanced CT technologies, including dual-energy CT and photon-counting detector CT, are of particular interest. Accumulating evidence indicates that spectral CT techniques can enhance lesion conspicuity and contrast-to-noise ratio, potentially allowing diagnostic performance comparable to conventional multiphasic protocols with fewer acquisitions or reduced radiation dose [32,33]. In addition, photon-counting detector CT combined with quantum iterative reconstruction has been shown to achieve substantial radiation dose reduction, reported up to approximately 50%, while maintaining or even improving liver lesion detection compared with conventional energy-integrating detector CT [29].

Beyond radiation dose considerations, spectral CT techniques may also facilitate the reduction in contrast media dose. Prospective randomized studies have demonstrated that low monoenergetic images, particularly around 50 keV, combined with advanced denoising or reconstruction strategies, allow for a 30–40% reduction in both radiation and contrast dose without compromising lesion detectability in patients at risk for HCC [34,35,36]. Deep learning-based reconstruction approaches have further been shown to preserve diagnostic image quality at approximately half the radiation dose compared with standard multiphasic protocols [37]. Particularly, the proof-of-concept study by Muenzel et al. demonstrated that the intrinsic spectral resolution of PCCT can be exploited to achieve simultaneous dual-phase liver imaging by sequential administration of iodine- and gadolinium-based contrast agents and material separation based on their distinct K-edge properties [24]. Similar concepts of single-acquisition multi-phase liver imaging have also been explored using spectral photon-counting CT with dual-contrast protocols [38]. Although these approaches are highly promising in terms of potential dose reduction and robustness to motion artifacts, they remain experimental and have not yet been validated in clinical HCC populations. Collectively, these findings suggest that spectral CT and photon-counting technologies hold substantial potential for reducing radiation and contrast dose, as well as for simplifying multiphasic imaging protocols. However, despite encouraging results from experimental and selected clinical studies, current evidence remains insufficient to support routine protocol simplification or dose reduction strategies in HCC imaging. Prospective studies employing standardized protocols and clinically meaningful endpoints are required to determine whether these technical advantages translate into improved patient outcomes.

### 4.4. Clinical Implications, Limitations, and Future Perspectives

Another relevant aspect emerging from the reviewed literature concerns the potential implications of PCCT for longitudinal imaging and follow-up in patients at risk for HCC. In clinical practice, patients with chronic liver disease often undergo repeated imaging examinations over prolonged periods, either for surveillance or post-treatment monitoring. In this context, cumulative radiation exposure, examination time, and robustness to motion artifacts represent critical considerations. Although none of the included studies specifically addressed longitudinal imaging strategies, the technical features of PCCT, such as improved dose efficiency, enhanced contrast resolution, and advanced reconstruction algorithms, may be particularly advantageous in scenarios requiring repeated assessments. Moreover, the improved spatial resolution achievable with photon-counting technology raises the question of whether PCCT could enhance the detection of subtle changes over time, including lesion growth, modifications in enhancement patterns, or treatment-related changes following locoregional therapies. Accurate longitudinal assessment is central to treatment response evaluation and retreatment planning in HCC management. However, current evidence does not yet support the application of PCCT for these purposes, and such potential advantages remain speculative in the absence of dedicated longitudinal or outcome-based studies.

Importantly, the integration of PCCT into routine clinical practice will depend on several practical and operational factors beyond image quality alone. Workflow efficiency, reconstruction time, data storage requirements associated with multi-energy datasets, and the learning curve required for interpretation of spectral PCCT images may influence adoption, particularly in high-volume clinical settings. In addition, standardization across vendors and platforms will be essential to ensure reproducibility and generalizability of results, yet this remains an unresolved issue given the early stage of clinical dissemination.

Despite encouraging technical developments, important limitations must be acknowledged. Available studies are small, heterogeneous, and predominantly focused on image quality rather than clinically meaningful endpoints. Evidence in cirrhotic patients remains limited. Furthermore, in the absence of direct comparative trials with established imaging modalities, any incremental advantage of PCCT over MRI or conventional CT remains hypothetical.

In conclusion, PCCT represents a promising multiparametric imaging approach with potential relevance for HCC imaging. However, current evidence is preliminary and largely focused on technical feasibility rather than diagnostic accuracy, clinical decision-making, or patient outcomes. Consequently, the role of PCCT in routine HCC imaging cannot yet be defined. Prospective, multicenter investigations with harmonized acquisition protocols, direct comparison with established imaging standards, and clinically meaningful endpoints, including diagnostic accuracy, workflow impact, radiation dose, and cost-effectiveness, will be required before PCCT can be considered for widespread clinical adoption.

## Figures and Tables

**Table 1:** Characteristics of the included articles.

First Author	Year of Publication	Study Aim	Patients	Dataset	Reference Standard	Results
Graafen [22]	2023	To identify the optimal reconstruction kernel for HCC imaging in a phantom and patient population	24 patients with HCC	VMIs at 50 keV with four sharpness levels (36–40–44–48)	N/A	Lower noise (18 ± 2) for a sharpness level of 36 (*p* < 0.001)
Estler [23]	2023	To compare arterial vs. portal venous polychromatic images with different VMIs for HCC detection	49 patients with HCC	VMIs 40–70 keV and polychromatic images (T3D)	MRI/CT follow-up	Accuracy 100%, best CNR at 40 keV, arterial phase ↑51% CTDI, ↑38% DLP without significant gain
Muenzel [24]	2017	Proof-of-concept of dual-contrast spectral PCCT for liver imaging	Simulation study based on CT data (1 healthy, 4 patients with lesions: HCC, haemangioma, cyst, metastasis)	Numerical spectral PCCT with sequential gadolinium (portal venous) + iodine (arterial) injections	Histology for HCC, metastasis/MRI follow-up for haemangioma, cyst	Spectral PCCT allowed for arterial vs. portal phases in a single scan
Szelenyi [25]	2025	To optimize the slice thickness and reconstruction kernel for PCCT in HCC	25 patients with HCC	Arterial-phase PCCT, multiple slice thicknesses (0.4, 1, 3 mm) and kernels (Br40, Br 44, Br48, Br 56)	Br40 kernel at 3 mm	-Br44/Br48 kernels improved vessel conspicuity; Br40 kernel provided the best overall lesion quality; -thinner slices (0.4 mm) increased noise and reduced quality
Graafen [26]	2023	To evaluate the effect of QIR on PCCT image quality for HCC	44 patients with cirrhosis and 75 HCC lesions	Triple-phase PCCCT at 50 keV reconstructed with FBP and QIR levels 1–4	Internal comparison across reconstruction levels (FBP vs. QIR)	QIR-4 gave the greatest noise suppression (−67% vs. FBP), best lesion conspicuity, and overall quality -progressive improvement from QIR-1 to QIR-4

HCC: hepatocellular carcinoma; VMI: virtual monoenergetic image; N/A: not applicable; CNR: contrast-to-noise ratio; PCCT: photon-counting computed tomography; QIR: quantitative iterative reconstruction; FBP: filtered back projection.

## Data Availability

No new data were created in this study.

## Supplementary Materials

The following supporting information can be downloaded at: https://www.mdpi.com/article/10.3390/diagnostics16050743/s1. Preferred Reporting Items for Systematic Reviews and Meta-Analyses (PRISMA) 2020 Checklist.
